# IK_Ca_-Ca_v_3 complex creates a high pass filter for parallel fiber input in cerebellar Purkinje cells

**DOI:** 10.1186/1471-2202-12-S1-P344

**Published:** 2011-07-18

**Authors:** Jordan DT Engbers, Dustin Anderson, Renata Rehak, Hamish W Mehaffey, Bruce E McKay, Mirna Kruskic, Gerald W Zamponi, Ray W Turner

**Affiliations:** 1 Hotchkiss Brain Institute, University of Calgary, Calgary, Alberta, Canada T2N 4N1

## 

Cerebellar Purkinje cells are contacted by up to ~150,000 parallel fibers from granule cells, of which only a subset will convey sensory information at any given time. Purkinje cells must then possess the means to respond effectively to meaningful parallel fiber input over background noise. Previous work has shown that parallel fiber excitatory postsynaptic potential (EPSP) summation can be shaped by feedforward synaptic inhibition and the hyperpolarization-activated current *I*_H_[1,2]. We now report that parallel fiber EPSPs activate T-type calcium channels that are linked to intermediate conductance calcium-activated potassium (IK_Ca_) channels in Purkinje cells. This novel complex exerts a frequency-dependent suppression of temporal summation, such that only high frequency parallel fiber inputs undergoing presynaptic facilitation can elicit spike output from Purkinje cells.

Cerebellar slices were prepared from P18-30 rats and patch recordings obtained from PC somata at 32-35°C. PFs were activated using a monopolar stimulating electrode in the molecular layer or granule cell layer. Alternatively, the role of postsynaptic PC ion channels were selectively tested by injecting simulated EPSCs to evoke PF simulated EPSPs (simEPSPs) at the soma.

PF EPSPs below threshold for spike discharge were followed by an after hyperpolarization (AHP) of up to 2.5 mV and 250 ms. Application of blockers against high voltage activated Ca^2+^ channels (Cd^2+^, Agatoxin IVA), SK channels (apamin), or BK channels (TEA, iberiotoxin, paxilline) did not significantly affect the rate of simEPSP decay. However, T-type Ca^2+^ channel blockers (Ni^2+^, Mibefradil, kurtoxin) caused a ~35% decrease in the rate of simEPSP decay. Moreover, these effects were reproduced by application of IK_Ca_ channel blockers (TRAM-34, charybdotoxin). Immunofluorescent labeling for IK_Ca_ protein confirmed its expression in Purkinje cells somata and dendrites. Ni^2+^ and TRAM-34 sensitive outward currents were found in outside-out patches from PC somata, confirming current clamp data showing a functional link between Ca_v_3 and IK_Ca_ channels. The outward current was further blocked by BAPTA (10 mM) but not EGTA (10 mM) in the internal patch solution, indicating that the Ca^2+^-IK_ca_ channel interaction resides within a nanodomain. To examine the effect of this interaction on temporal summation, PFs were stimulated at varying frequencies. For frequencies up to 25 Hz, no temporal summation was observed in control conditions. However, blocking either Ca_v_3 or IK_ca_ channels caused significant summation for 25 Hz stimulations. This effect was seen in both the presence and absence of feedforward-inhibition. Application of TRAM-34 greatly altered the frequency response of PC to 50 and 100 Hz PF stimulation during tonic firing. Finally, the Ca_v_3-IK_Ca_ complex selectively suppresses non-facilitating inputs while allowing smaller-amplitude, facilitating inputs to generate output.

Our current work is the first to demonstrate the expression of IK_Ca_ channels in central neurons, its association with Cav3 channels and the role of this Cav3-IKCa complex in controlling the response of PCs to PF inputs. The Cav3-IKCa complex creates a high pass filter that reduces the effectiveness of background activity and allows Purkinje cells to respond preferentially to parallel fiber input indicative of sensory input carried by mossy fibers.

## References

[B1] Angelo K London M Christensen SR Hausser M Local and global effects of IH distribution in dendrites of mammalian neurons J Neurosci 2007 27 32 8643 8653 10.1523/JNEUROSCI.5284-06.2007 17687042 PMC6672943

[B2] Mittmann W Koch U Hausser H Feed-forward inhibition shapes the spike output of cerebellar Purkinje cells J Physiol 2005 563 2 369 378 15613376 10.1113/jphysiol.2004.075028 PMC1665592

